# Supplementary material for: Multi‐dataset identification of innovative feature genes and molecular mechanisms in keratoconus
Source: J Cell Mol Med. 2024 Sep 19;28(18):e70079. doi: 10.1111/jcmm.70079 (PMC11412914; doi:10.1111/jcmm.70079)
Supplement: Supplementary file 2 — Material S2. [file JCMM-28-e70079-s002.zip › 7.GSEA analysis/4.MYRF/KEGG_CELL_ADHESION_MOLECULES_CAMS.html]

Details for gene set KEGG\_CELL\_ADHESION\_MOLECULES\_CAMS[GSEA]

|  || Dataset | MYRF.group.cls#h\_versus\_l |
| Phenotype | group.cls#h\_versus\_l |
| Upregulated in class | l |
| GeneSet | KEGG\_CELL\_ADHESION\_MOLECULES\_CAMS |
| Enrichment Score (ES) | -0.47375873 |
| Normalized Enrichment Score (NES) | -1.5315012 |
| Nominal p-value | 0.012219959 |
| FDR q-value | 0.21595477 |
| FWER p-Value | 0.713 |
Table: GSEA Results Summary

  

Fig 1: Enrichment plot: KEGG\_CELL\_ADHESION\_MOLECULES\_CAMS      
 Profile of the Running ES Score & Positions of GeneSet Members on the Rank Ordered List

  

| SYMBOL | TITLE | RANK IN GENE LIST | RANK METRIC SCORE | RUNNING ES | CORE ENRICHMENT || 1 | CLDN22 | na | 211 | 0.707 | 0.0213 | No |
| 2 | CD86 | na | 355 | 0.626 | 0.0415 | No |
| 3 | SELP | na | 614 | 0.541 | 0.0550 | No |
| 4 | ITGA4 | na | 1618 | 0.389 | 0.0411 | No |
| 5 | ITGAL | na | 2764 | 0.308 | 0.0200 | No |
| 6 | CLDN5 | na | 3852 | 0.259 | -0.0014 | No |
| 7 | CD22 | na | 4222 | 0.246 | -0.0025 | No |
| 8 | ICAM2 | na | 5490 | 0.208 | -0.0310 | No |
| 9 | CD2 | na | 5706 | 0.201 | -0.0294 | No |
| 10 | ITGA9 | na | 5848 | 0.197 | -0.0259 | No |
| 11 | NLGN3 | na | 6207 | 0.186 | -0.0290 | No |
| 12 | CLDN18 | na | 6240 | 0.185 | -0.0228 | No |
| 13 | VCAM1 | na | 7772 | 0.144 | -0.0615 | No |
| 14 | ICOSLG | na | 7776 | 0.144 | -0.0560 | No |
| 15 | HLA-DOA | na | 8167 | 0.134 | -0.0620 | No |
| 16 | CLDN20 | na | 8679 | 0.123 | -0.0720 | No |
| 17 | MPZ | na | 9135 | 0.113 | -0.0808 | No |
| 18 | ITGB7 | na | 9321 | 0.109 | -0.0819 | No |
| 19 | CLDN7 | na | 9372 | 0.108 | -0.0791 | No |
| 20 | CLDN3 | na | 9491 | 0.106 | -0.0784 | No |
| 21 | PDCD1LG2 | na | 9695 | 0.102 | -0.0804 | No |
| 22 | SELPLG | na | 9891 | 0.098 | -0.0822 | No |
| 23 | CD4 | na | 10052 | 0.095 | -0.0831 | No |
| 24 | NLGN1 | na | 10146 | 0.093 | -0.0822 | No |
| 25 | SDC3 | na | 10402 | 0.089 | -0.0861 | No |
| 26 | OCLN | na | 10725 | 0.083 | -0.0922 | No |
| 27 | ICAM3 | na | 11343 | 0.073 | -0.1072 | No |
| 28 | VCAN | na | 11436 | 0.071 | -0.1071 | No |
| 29 | ICOS | na | 11751 | 0.067 | -0.1136 | No |
| 30 | NLGN4X | na | 12261 | 0.060 | -0.1260 | No |
| 31 | CD28 | na | 12283 | 0.059 | -0.1243 | No |
| 32 | CLDN19 | na | 12427 | 0.058 | -0.1262 | No |
| 33 | CLDN16 | na | 12551 | 0.056 | -0.1276 | No |
| 34 | CLDN23 | na | 12578 | 0.056 | -0.1262 | No |
| 35 | SPN | na | 12903 | 0.051 | -0.1335 | No |
| 36 | CLDN8 | na | 12946 | 0.051 | -0.1328 | No |
| 37 | HLA-B | na | 12962 | 0.051 | -0.1312 | No |
| 38 | NEGR1 | na | 13320 | 0.046 | -0.1398 | No |
| 39 | PDCD1 | na | 13374 | 0.045 | -0.1396 | No |
| 40 | SDC2 | na | 13855 | 0.040 | -0.1519 | No |
| 41 | CNTN1 | na | 13937 | 0.039 | -0.1527 | No |
| 42 | PTPRF | na | 14783 | 0.030 | -0.1760 | No |
| 43 | SDC1 | na | 14892 | 0.029 | -0.1780 | No |
| 44 | CDH1 | na | 15708 | 0.021 | -0.2008 | No |
| 45 | MPZL1 | na | 15932 | 0.018 | -0.2065 | No |
| 46 | CNTN2 | na | 16188 | 0.016 | -0.2132 | No |
| 47 | SELE | na | 16216 | 0.016 | -0.2134 | No |
| 48 | HLA-F | na | 16261 | 0.015 | -0.2141 | No |
| 49 | HLA-E | na | 16263 | 0.015 | -0.2136 | No |
| 50 | MAG | na | 16779 | 0.010 | -0.2280 | No |
| 51 | HLA-C | na | 17654 | 0.003 | -0.2532 | No |
| 52 | CLDN9 | na | 18587 | -0.006 | -0.2799 | No |
| 53 | F11R | na | 18606 | -0.006 | -0.2802 | No |
| 54 | NEO1 | na | 18817 | -0.008 | -0.2859 | No |
| 55 | ALCAM | na | 19498 | -0.016 | -0.3049 | No |
| 56 | CADM1 | na | 19508 | -0.016 | -0.3046 | No |
| 57 | HLA-A | na | 20084 | -0.023 | -0.3203 | No |
| 58 | HLA-DRB5 | na | 20183 | -0.024 | -0.3222 | No |
| 59 | HLA-G | na | 20210 | -0.024 | -0.3220 | No |
| 60 | NLGN2 | na | 20943 | -0.033 | -0.3419 | No |
| 61 | ITGB2 | na | 21824 | -0.044 | -0.3656 | No |
| 62 | GLG1 | na | 22333 | -0.050 | -0.3784 | No |
| 63 | CNTNAP1 | na | 22683 | -0.055 | -0.3863 | No |
| 64 | CD99 | na | 22828 | -0.057 | -0.3883 | No |
| 65 | CD8B | na | 23062 | -0.061 | -0.3927 | No |
| 66 | HLA-DRB3 | na | 23156 | -0.062 | -0.3930 | No |
| 67 | CD276 | na | 23364 | -0.065 | -0.3964 | No |
| 68 | CLDN1 | na | 23552 | -0.068 | -0.3992 | No |
| 69 | CTLA4 | na | 23769 | -0.072 | -0.4026 | No |
| 70 | SDC4 | na | 23814 | -0.073 | -0.4011 | No |
| 71 | CDH5 | na | 23848 | -0.073 | -0.3992 | No |
| 72 | HLA-DQB1 | na | 24038 | -0.076 | -0.4017 | No |
| 73 | HLA-DRB1 | na | 24046 | -0.077 | -0.3989 | No |
| 74 | HLA-DOB | na | 24105 | -0.078 | -0.3976 | No |
| 75 | CDH4 | na | 24380 | -0.082 | -0.4023 | No |
| 76 | PTPRC | na | 24862 | -0.091 | -0.4127 | No |
| 77 | CD58 | na | 25040 | -0.094 | -0.4142 | No |
| 78 | NRXN2 | na | 25164 | -0.096 | -0.4140 | No |
| 79 | CD8A | na | 25192 | -0.097 | -0.4110 | No |
| 80 | CLDN4 | na | 25502 | -0.103 | -0.4160 | No |
| 81 | CD80 | na | 26452 | -0.122 | -0.4387 | No |
| 82 | L1CAM | na | 26845 | -0.131 | -0.4449 | No |
| 83 | ITGAV | na | 26862 | -0.132 | -0.4402 | No |
| 84 | CLDN15 | na | 27173 | -0.139 | -0.4438 | No |
| 85 | CLDN11 | na | 27203 | -0.140 | -0.4392 | No |
| 86 | ESAM | na | 27456 | -0.147 | -0.4408 | No |
| 87 | HLA-DMA | na | 27498 | -0.148 | -0.4363 | No |
| 88 | CLDN10 | na | 28723 | -0.182 | -0.4646 | No |
| 89 | CDH3 | na | 28912 | -0.188 | -0.4627 | No |
| 90 | CNTNAP2 | na | 29296 | -0.200 | -0.4660 | Yes |
| 91 | ITGA8 | na | 29346 | -0.201 | -0.4596 | Yes |
| 92 | ITGA6 | na | 29414 | -0.203 | -0.4537 | Yes |
| 93 | PTPRM | na | 29667 | -0.212 | -0.4527 | Yes |
| 94 | ITGB1 | na | 29873 | -0.219 | -0.4501 | Yes |
| 95 | HLA-DRB4 | na | 30247 | -0.232 | -0.4519 | Yes |
| 96 | NRCAM | na | 30315 | -0.234 | -0.4448 | Yes |
| 97 | CDH2 | na | 30517 | -0.243 | -0.4411 | Yes |
| 98 | SELL | na | 30637 | -0.248 | -0.4350 | Yes |
| 99 | CD40LG | na | 30708 | -0.251 | -0.4273 | Yes |
| 100 | ITGB8 | na | 30931 | -0.260 | -0.4236 | Yes |
| 101 | CD6 | na | 31293 | -0.280 | -0.4232 | Yes |
| 102 | SIGLEC1 | na | 31485 | -0.290 | -0.4174 | Yes |
| 103 | CLDN17 | na | 31502 | -0.291 | -0.4066 | Yes |
| 104 | PECAM1 | na | 31598 | -0.295 | -0.3979 | Yes |
| 105 | ITGAM | na | 31711 | -0.302 | -0.3894 | Yes |
| 106 | PVR | na | 31763 | -0.305 | -0.3791 | Yes |
| 107 | HLA-DPA1 | na | 32053 | -0.320 | -0.3750 | Yes |
| 108 | CD226 | na | 32385 | -0.342 | -0.3713 | Yes |
| 109 | CLDN6 | na | 32462 | -0.348 | -0.3600 | Yes |
| 110 | NFASC | na | 32493 | -0.350 | -0.3473 | Yes |
| 111 | CD40 | na | 32572 | -0.356 | -0.3357 | Yes |
| 112 | NCAM1 | na | 32848 | -0.376 | -0.3291 | Yes |
| 113 | NRXN1 | na | 32880 | -0.378 | -0.3153 | Yes |
| 114 | HLA-DRA | na | 33031 | -0.390 | -0.3045 | Yes |
| 115 | NCAM2 | na | 33101 | -0.397 | -0.2911 | Yes |
| 116 | HLA-DMB | na | 33256 | -0.414 | -0.2795 | Yes |
| 117 | CADM3 | na | 33310 | -0.420 | -0.2647 | Yes |
| 118 | CD274 | na | 33464 | -0.438 | -0.2521 | Yes |
| 119 | MADCAM1 | na | 33498 | -0.443 | -0.2359 | Yes |
| 120 | CD34 | na | 33942 | -0.511 | -0.2289 | Yes |
| 121 | ICAM1 | na | 34123 | -0.548 | -0.2128 | Yes |
| 122 | CLDN2 | na | 34148 | -0.557 | -0.1919 | Yes |
| 123 | CDH15 | na | 34197 | -0.570 | -0.1711 | Yes |
| 124 | HLA-DQA2 | na | 34217 | -0.576 | -0.1493 | Yes |
| 125 | JAM2 | na | 34223 | -0.578 | -0.1270 | Yes |
| 126 | CLDN14 | na | 34256 | -0.588 | -0.1051 | Yes |
| 127 | HLA-DPB1 | na | 34331 | -0.609 | -0.0836 | Yes |
| 128 | NRXN3 | na | 34490 | -0.673 | -0.0621 | Yes |
| 129 | HLA-DQA1 | na | 34602 | -0.745 | -0.0364 | Yes |
| 130 | JAM3 | na | 34714 | -1.030 | 0.0004 | Yes |
Table: GSEA details [plain text format]

  

Fig 2: KEGG\_CELL\_ADHESION\_MOLECULES\_CAMS      
 Blue-Pink O' Gram in the Space of the Analyzed GeneSet

  

Fig 3: KEGG\_CELL\_ADHESION\_MOLECULES\_CAMS: Random ES distribution      
 Gene set null distribution of ES for **KEGG\_CELL\_ADHESION\_MOLECULES\_CAMS**
